# Eps 15 Homology Domain (EHD)-1 Remodels Transverse Tubules in Skeletal Muscle

**DOI:** 10.1371/journal.pone.0136679

**Published:** 2015-09-01

**Authors:** Alexis R. Demonbreun, Kaitlin E. Swanson, Ann E. Rossi, H. Kieran Deveaux, Judy U. Earley, Madison V. Allen, Priyanka Arya, Sohinee Bhattacharyya, Hamid Band, Peter Pytel, Elizabeth M. McNally

**Affiliations:** 1 Center for Genetic Medicine, Northwestern University, Chicago, IL, United States of America; 2 Department of Pathology, The University of Chicago, Chicago, IL, United States of America; 3 Department of Medicine, The University of Chicago, Chicago, IL, United States of America; 4 Department of Genetics, Cell Biology & Anatomy, University of Nebraska Medical Center, Omaha, NE, United States of America; 5 Department of Pathology & Microbiology, Eppley Institute for Research in Cancer and Allied Diseases, University of Nebraska Medical Center, Omaha, NE, United States of America; University of Debrecen, HUNGARY

## Abstract

We previously showed that Eps15 homology domain-containing 1 (EHD1) interacts with ferlin proteins to regulate endocytic recycling. Myoblasts from *Ehd1*-null mice were found to have defective recycling, myoblast fusion, and consequently smaller muscles. When expressed in C2C12 cells, an ATPase dead-EHD1 was found to interfere with BIN1/amphiphysin 2. We now extended those findings by examining *Ehd1*-heterozygous mice since these mice survive to maturity in normal Mendelian numbers and provide a ready source of mature muscle. We found that heterozygosity of EHD1 was sufficient to produce ectopic and excessive T-tubules, including large intracellular aggregates that contained BIN1. The disorganized T-tubule structures in *Ehd1*-heterozygous muscle were accompanied by marked elevation of the T-tubule-associated protein DHPR and reduction of the triad linker protein junctophilin 2, reflecting defective triads. Consistent with this, *Ehd1*-heterozygous muscle had reduced force production. Introduction of ATPase dead-EHD1 into mature muscle fibers was sufficient to induce ectopic T-tubule formation, seen as large BIN1 positive structures throughout the muscle. *Ehd1*-heterozygous mice were found to have strikingly elevated serum creatine kinase and smaller myofibers, but did not display findings of muscular dystrophy. These data indicate that EHD1 regulates the maintenance of T-tubules through its interaction with BIN1 and links T-tubules defects with elevated creatine kinase and myopathy.

## Introduction

Loss of function mutations in *BIN1*, the gene encoding the membrane trafficking protein BIN1/amphiphysin 2, cause severe forms of myopathy with muscle weakness evident at birth [1,2]. Autosomal dominant mutations in *DNM2*, the gene encoding dynamin 2, another membrane trafficking protein, also cause myopathy resulting in both mild and severe forms of disease [3,4]. Dynamins are large GTPases that alter actin dynamics and membrane trafficking by forming rings encasing membrane tubules and aiding in the fission process. Although dynamin is ubiquitously expressed, *DNM2* mutations manifest with muscle weakness presumably through gain-of-function activity that targets vesicles and membrane remodeling in muscle. Recently, it has been shown that partial reduction of dynamin 2 protein was sufficient to rescue the muscle pathology of myotubularin-deficient mice, suggesting a potential therapeutic role for this large family of proteins [5]. Myotubularin is a phosphoinositide phosphatase, and loss of function *MTM1* mutations also cause myopathy [1].

Structural studies have shown a similarity between the G-domain of Eps15 Homology Domain (EHD) proteins and dynamin, predicting a “pinchase” role for EHDs in regulating membrane fission by assembling into spiral rings. This function was confirmed by studies with lamprey EHD, l-EHD, in which l-EHD strongly inhibited excessive assembly of dynamin and the formation of elongated vesicular structures [6]. Distinct from dynamin, EHDs directly associate with other proteins through the EH domain which binds with proteins containing the asparagine-proline-phenylalanine (NPF) motif typically followed by acidic residues such as aspartic acid and/or glutamic acid [7]. This specific binding sequence is found in EHD binding partners such as BIN1, Rab-interacting proteins, and the ferlin family proteins myoferlin and Fer1L5. All of these proteins are implicated in aspects of vesicle trafficking and recycling [8,9].

The EHD family of proteins regulates multiple steps of endocytic and vesicle trafficking [10,11]. The mammalian EHD family consists of four proteins, EHD1-4, while there is a single EHD protein in both *Drosophila* (PAST-1) and *C*. *elegans* (RME-1). The EHD proteins contain a P-loop within the N-terminal G-domain that hydrolyzes ATP, a central helical region, and a C-terminal EH domain. Mutations in the P-loop or coiled-coil region have been shown to interfere with ATP hydrolysis and alter oligomerization, a property essential for function [12]. Despite the absence of a transmembrane domain, EHD family members associate with the membrane of vesicles and tubular structures. The ability of EHDs to associate with and form membrane-bound tubules requires the P-loop, oligomerization, and protein/protein interaction domains. BIN1 coimmunoprecipitates and colocalizes with EHD1, and EHD1 functions in conjunction with BIN1 to refine the length and width of membrane tubules [9,13]. EHD family members are highly related, approximately 70% between EHD1 and EHD2 and 86% between EHD1 and EHD3, although patterns of tissue expression are distinct.

During muscle development, EHD proteins are differentially expressed. EHD2 is expressed early in myoblasts before fusion to multinucleate myotubes, while EHD1 and EHD4 are expressed during myotube formation and maturation [8]. In myotubes that have been wounded, EHD2 is recruited to the site of sarcolemmal damage [11]. In the failing heart, EHD3 levels are increased, suggesting a role for EHD3 in the remodeling cardiomyocyte [14]. Mature skeletal muscle relies on deep membrane invaginations, referred to as transverse, T-tubules, in order to uniformly trigger rapid Ca^2+^ release throughout the myofiber and orchestrate muscle contraction [15–17]. The remodeling of T-tubules in mature myofibers is not well understood, but the presence of a rich T-tubule network provides the normal intracellular localization to scaffold Ca^2+^-handling proteins including the ryanodine receptor and other Ca^2+^ channels [16,18,19].

Because EHD1 is expressed in muscle and regulates membrane trafficking, we examined mice heterozygous for an *Ehd1* gene deletion that produces partial reduction of EHD1 [8,13,20]. *Ehd1-*heterozygous muscle was associated with elongated and ectopic T-tubule structures in skeletal muscle and reduced force production *ex vivo*. T-tubule associated Ca^2+^-handling proteins, including the dihydropyridine receptor (DHPR) and junctophilin, were mis-expressed with increased DHPR and reduced junctophilin in *Ehd1-*heterozygous muscle. The disrupted expression of Ca^2+^-handling proteins may explain the observed muscle weakness in *Ehd1-*heterozygous muscle. Expression of dominant-negative EHD1 in muscle was found to induce similar T-tubule defects with aggregation and excessive accumulation, suggesting that EHD1 acts to negatively remodel the T-tubule.

## Results

### Transverse-tubule abnormalities in *Ehd1-*heterozygous muscle

The *Ehd1*-null allele was previously generated, and most homozygous null *EHD1* mice die *in utero* or at birth [20,21]. The surviving mice develop male infertility [13,20]. We previously examined myoblasts from *Ehd1* null mice finding evidence for delayed endocytic recycling and smaller myofibers [13]. Using a cell culture system, we found evidence for an interaction between BIN1 and EHD1, and in the few surviving mice, complete loss of EHD1 in *Ehd1*-null mice resulted in disorganized T-tubules [13].

**Fig 1:**
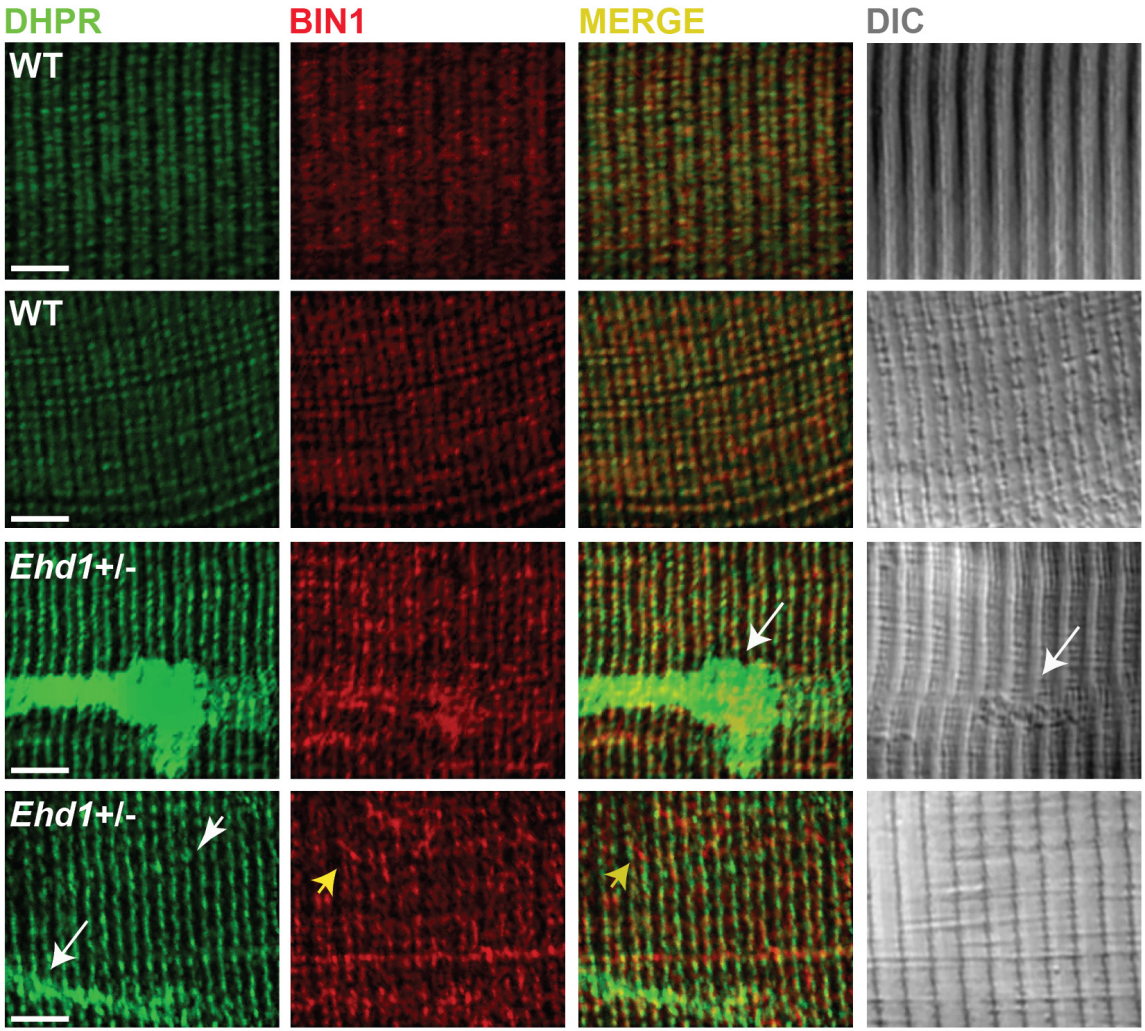
Disordered T-tubules in *Ehd1-*heterozygous muscle. Myofibers were immunostained with anti-BIN1 (red) and anti-DHPR (green) antibodies. Representative myofibers are shown. *Ehd1-*heterozygous (*Ehd1+/-*) fibers displayed disorganized (white arrowhead) and aggregated (white arrow) T-tubule structures in 27% of myofibers, marked by DHPR, also evidenced in DIC images compared to 0% in control fibers. *Ehd1*-heterozygous muscle with extensive BIN1 fluorescence extending beyond DHPR staining (yellow arrowhead). Scale 5μm.

BIN1 normally is essential for normal T-tubule development in muscle helps tether DHPR to the T-tubule [13,22–25]. To determine whether endogenous BIN1 was altered in *Ehd1-*heterozygous muscle, myofibers were isolated and stained with antibodies to BIN1 and DHPR. *Ehd1-*heterozygous fibers displayed disordered T-tubules marked by BIN1 and DHPR fluorescence (Fig 1). The DHPR puncta were highly disorganized with ectopic T-tubules (Fig 1, white arrowhead), similar to the disorganized T-tubule pattern seen in muscle from dysferlin-null mice, a model of Limb girdle muscular dystrophy 2B [22]. High magnification imaging showed BIN1 elongation that extended beyond the DHPR domain (Fig 1, yellow arrowhead), consistent with expansion of the membranous network. Wildtype T-tubules, marked by BIN1 and DHPR fluorescence, were found in the expected ordered pattern, with rare instances of elongated BIN1 fluorescence (Fig 1). Large aggregates of T-tubules were visualized in *Ehd1-*heterozygous fibers, with 27% of fibers showing this pattern (3/11 fibers). These aggregates were positive for DHPR and BIN1, and this compared to 0% of aggregates in control, wildtype muscle fibers (0/9 fibers) (Fig 1, white arrow).

**Fig 2:**
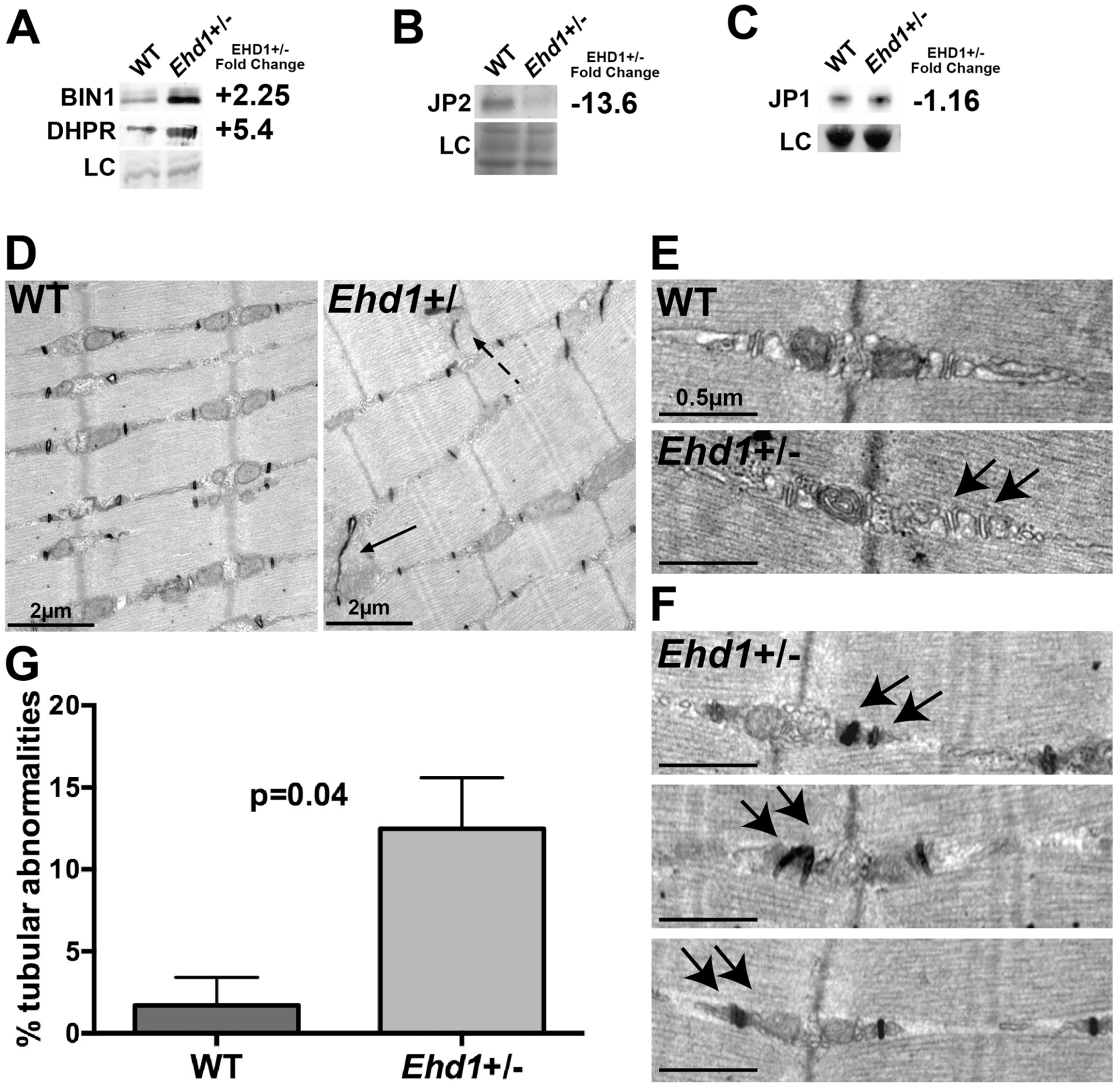
Misexpression of triad proteins and expansion of the T-tubule compartment in *Ehd1-*heterozygous muscle. (A) BIN1 and DHPR levels are increased +2.25 and +5.4 fold in *Ehd1*+/- quadriceps muscle compared to WT correlating with the increase in T-tubule structures. Gel code bands are shown as a loading control (LC). (B) Junctophilin 2 (JP2) protein levels were decreased 13-fold in *Ehd1+/-* quadriceps muscle compared to wildtype controls. Gel code stained bands are shown as a loading control (LC). (C) Junctophilin 1 (JP1) protein levels were similar in *Ehd1+/-* quadriceps muscle compared to wildtype controls. Gel code stained bands are shown as a loading control (LC). (D) Ultrastructural analysis reveals ectopic (dotted arrow) and elongated (arrow) T-tubules in 8-week-old *Ehd1-*heterozygous muscle (*Ehd1+/-*) stained with potassium ferricyanide to color the T-tubule structures black. (E) *Ehd1-*heterozygous muscle contains duplicated triads containing 2 T-tubules (black arrows) and 3 sarcoplasmic reticulum (SR) in 1 triad unit. Scale 0.5μm. (F) *Ehd1-*heterozygous muscle stained with potassium ferricyanide, outlines duplicated T-tubule structures (two black arrows). Scale 0.5μm. (G) Ultrastructural analysis of 2-D images reveals increased tubule abnormalities in *Ehd1-*heterozygous muscle, 12.5%, compared to 1.7% in control muscle (n>400 structures per genotype, p = 0.04).

Ca^2+^-handling proteins are enriched in T-tubules, and DHPR is one of the major proteins that regulate Ca^2+^ release to coordinate muscle contraction [19,23]. Immunoblot and density analysis revealed *Ehd1-*heterozygous muscle showed an increase of both BIN1 (+2.25 fold), and DHPR (+5.4 fold) protein levels compared to WT (Fig 2A, n≥3 of each genotype). Junctophilins span the endoplasmic reticulum (ER) / sarcoplasmic reticulum (SR) membranes to stabilize T-tubules and to facilitate attachment to the plasma membrane [26]. Loss of JP1 or JP2 in mice results in disorganized triads and reduced Ca^2+^ homeostasis and ultimately embryonic lethality [26,27]. *Ehd1-*heterozygous skeletal muscle had a 13-fold decrease of JP2 protein compared to controls (Fig 2B, n≥3 of each genotype) while JP1 protein expression was unchanged between *Ehd1-*heterozygous skeletal muscle and controls (Fig 2C, n = 2 of each genotype). The increased expression of BIN1 and DHPR in addition to the reduction of JP2 in *Ehd1-*heterozygous mice correlates with disorganization of the T-tubule system. These data provide evidence of a molecular network that functions to control T-tubule organization and is dysregulated in *Ehd1-*heterozygous mice.

### Ultrastructural T-tubule overgrowth in *Ehd1-*heterozygous muscle

T-tubule abnormalities are seen in multiple myopathic states, including the central nuclear myopathies and dysferlin-mediated myopathies [1,2,28–31]. EHD1 regulates the formation of small intracellular tubules in cell culture, and complete loss of *Ehd1* produced T-tubule elongation [13]. Ultrastructural analysis of *Ehd1*-heterozygous muscle displayed similarly elongated (Fig 2D, black arrow) and ectopic (Fig 2D, black dotted arrow) T-tubules, indicating that partial reduction of EHD1 is sufficient to cause this effect. In mammalian muscle sarcomeres, a triad is composed of a T-tubule centered between two terminal cisternae of sarcoplasmic reticulum (SR). Magnified ultrastructural images of *Ehd1*-heterozygous muscle showed duplicated triad structures, containing additional T-tubule and SR units (Fig 2E). Additional images from *Ehd1*-heterozygous muscle stained with potassium ferricyanide, which colors the T-tubules black, outlined the duplicated T-tubule structures seen within a single triad (Fig 2F). T-tubule abnormalities were quantified from 2-D ultrastructural images. *Ehd1*-heterozygous muscle showed an increase in tubule structure abnormalities, 12.5%, compared to 1.7% in control muscle (Fig 2G, p = 0.04, >400 structures analyzed per genotype). This data indicates that even partial reduction of EHD1 is sufficient for T-tubule remodeling.

### Reduced force production in *Ehd1-*heterozygous muscle

**Fig 3:**
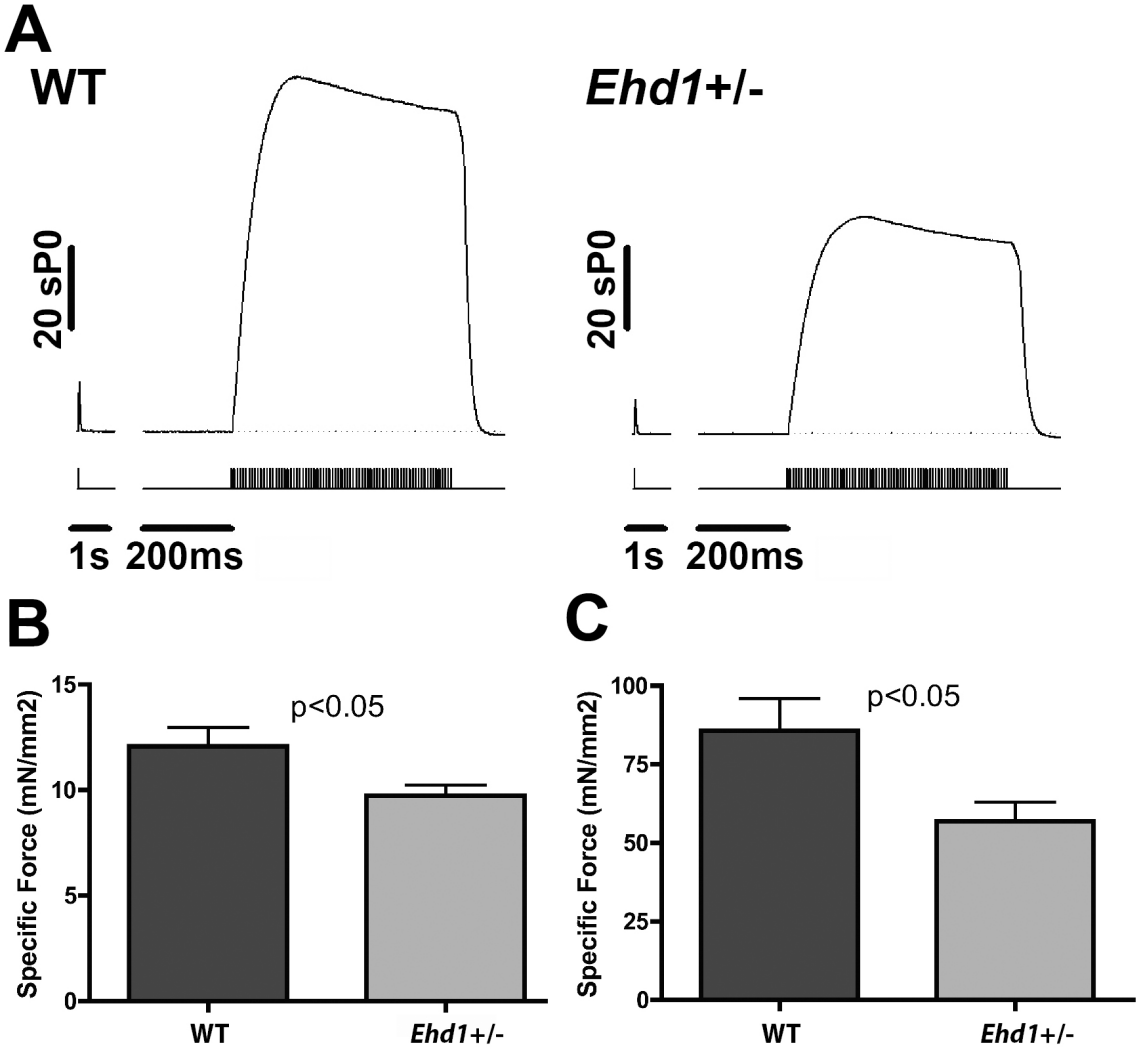
Reduction in EDL force production in *Ehd1-*heterozygous muscle. (A) Representative traces from 8-week male WT and *Ehd1-*heterozygous (*Ehd1+/-)* EDL muscles with stimulation pulses marked below the force traces. *Ehd1+/-* muscle has reduced force. (B) *Ehd1+/-* EDL muscle has reduced twitch force (n > 5 per genotype, p<0.05). (C) *Ehd1+/-* EDL muscle has reduced maximum tetanic force (n > 5 per genotype, p = 0.05).

To assess muscle function *ex vivo*, muscle mechanical measurements were using the *extensor digitorum longus* (EDL) muscle using a force transducer. Representative force tracings from 8-week-old wildtype and *Ehd1-*heterozygous mice showed reduced specific force production in *Ehd1-*heterozygous muscles (Fig 3A). Twitch force in *Ehd1-*heterozygous EDLs (9.73 mN/mm^2^) was reduced compared to wildtype (12.08 mN/mm^2^) (Fig 3B, n = 5 mice each, p<0.05). Maximal tetanic force production in *Ehd1-*heterozygous (56.92 mN/mm^2^) muscle was significantly reduced compared to wildtype (85.72 mN/mm^2^) (Fig 3C, n = 5 mice each, p<0.05). These data show that reduction of EHD1 is sufficient to cause appreciable muscle weakness in mice.

### *Ehd1-*heterozygous mice have myopathy but not dystrophy

It was shown previously that the complete genetic loss of *Ehd1* ablated EHD1 protein expression in multiple tissues including heart and skeletal muscle [20,21]. Intermediate levels of EHD1 were evident by immunoblot in *Ehd1*-heterozgous cardiac muscle [20]. Skeletal muscle from 8–12 week old wildtype and *Ehd1*-heterozygous mice was assessed by immunoblot utilizing antibody against EHD1 (S1A Fig). Gelcode was used as a loading control. *Ehd1*-heterozygous skeletal muscle has a reduction in EHD1 protein level (30% compared to wildtype 88% p = 0.003, n = 4 muscles per genotype).

The pathway of muscle degeneration and regeneration is often characterized by discrete histological features including internalization of nuclei, representative of myofiber repair after injury, increased fatty and immune infiltration, and abnormal fiber distribution. In progressive muscular dystrophy, these factors increase over time, as myofiber degeneration outpaces myofiber repair. To determine if *Ehd1-*heterozygous mice display signs of muscular dystrophy, due to the decreased levels of EHD1, we evaluated the muscle histopathology of both young and aged mice. *Ehd1-*heterozygous muscles contained multiple small myofibers versus age-matched wildtype controls, indicative of fiber splitting and/or regeneration (S1B Fig). The mean cross-sectional area (CSA) of *Ehd1-*heterozygous myofibers was reduced in muscles from young animals (S1C Fig, p = 0.003 for triceps muscles). We examined the distribution of myofiber size in muscle and found that 8-week-old *Ehd1-*heterozygous muscle specifically lacked the largest myofibers and contained an increased number of smaller myofibers (S1D Fig). To assess this distribution, we compared variances utilizing an F-test and determined that there was an unequal distribution of fibers (p<0.0001). Internal nuclei were rarely seen in *Ehd1-*heterozygous triceps muscle and WT controls at 8-weeks of age (p = 0.64, n = 3 mice per genotype, n>1000 fibers). There was no evidence of fibrosis or fatty infiltration seen in muscular dystrophy in young or old animals (S1E Fig).

### *Ehd1-*heterozygous mice have significantly elevated serum creatine kinase

**Fig 4:**
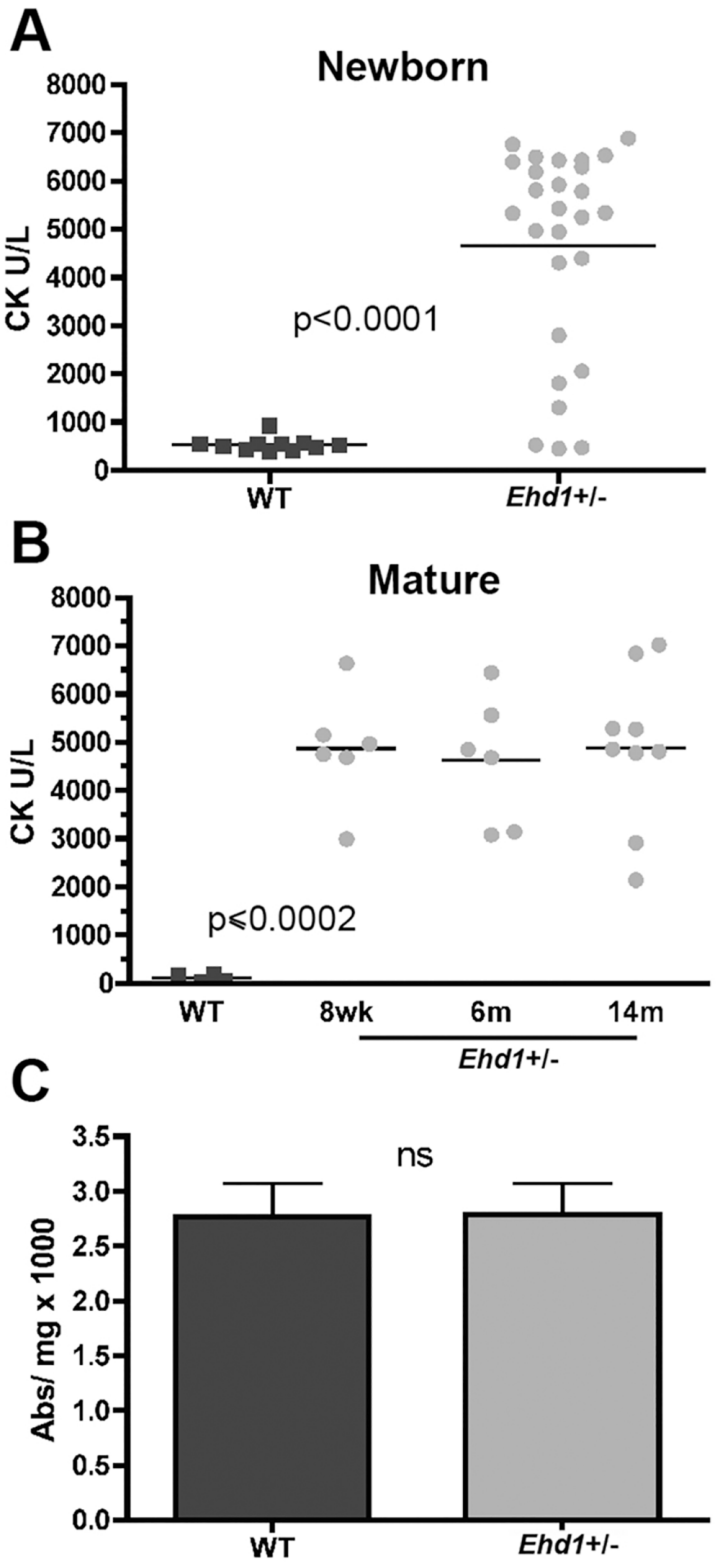
*Ehd1-*heterozygous mice have elevated creatine kinase levels. (A) Serum creatine kinase (CK) levels are highly elevated in *Ehd1-*heterozygous (*Ehd1+/-*) mice at birth (n > 10 of each genotype, p<0.0001). (B) CK levels are consistently elevated in *Ehd1+/-* mice at 8wk, 6m, and 14m compared to WT controls (n ≥ 6 of each genotype, p<0.0002). (C) Evans blue dye uptake was measured from excised tissues (absorbance per mg tissue). Graph expressed as an average of all tissues. Evans blue dye uptake (measured as absorbance) is similar in *Ehd1+/-* and WT (n>6 animals per genotype, ns, nonsignificant).

Creatine kinase (CK) generates phosphocreatine to allow more ready use of ATP in high energy demand cells like myofibers. CK leaks from muscle into the serum after intense exercise or injury, or in muscle disease [32]. Interestingly, serum CK levels were markedly elevated at birth (P0) in *Ehd1-*heterozygous mice (*Ehd1+/-* 4641 U/L vs WT controls 532 U/L, p<0.0001) (Fig 4A). Serum CK levels were persistently elevated in *Ehd1-*heterozygous mice throughout their lives with CK levels more than 40-fold higher than wildtype controls at all ages analyzed (n = 4 WT, n = 6 *Ehd1+/-*, p< 0.002) (Fig 4B). Blood urea nitrogen (BUN) and creatinine levels were normal in *Ehd1-*heterozygous mice, consistent with normal clearance through the kidney (BUN for *Ehd1-*heterozygous was 32.4 mg/dL versus 30.4 mg/dL for wildtype, n = 5 WT, n = 4 *Ehd1+/-*, p = 0.7; creatinine for *Ehd1-*heterozygous was 0.23 mg/dL compared to 0.26 mg/dL for wildtype controls, n = 3 WT, n = 2 *Ehd1+/-*, p = 0.4). These data indicate a muscle origin for serum CK elevation occurring in the absence of overt histopathological changes.

CK and other muscle proteins are hypothesized to leak into the serum with sarcolemmal disruption [33]. Sarcolemmal disruption can also be monitored by measuring uptake of vital tracers such as the small molecular mass marker Evans blue dye [34]. Eight-week-old wildtype and *Ehd1-*heterozygous mice were injected with dye and muscles were harvested for analysis. WT and *Ehd1-*heterozygous muscle displayed similar levels of dye uptake (Fig 4C). There was no significant difference in the dye uptake of any muscle group assayed (quadriceps (p = 0.92), gluteus/hamstring (p = 0.80), triceps (p = 0.66), gastrocnemius/soleus (p = 0.75), abdominal muscles (p = 0.81), and diaphragm (p = 0.98) between *Ehd1-*heterozygous muscle compared to wildtype controls (n = 6 WT, n = 7 *Ehd1+/-*) (S2 Fig). The absence of muscle breakdown in *Ehd1*-heterozygous muscle in the presence of markedly elevated serum CK links T-tubule defects with CK release.

### Functional EHD1 is required for proper BIN1 localization and T-tubule formation in vivo

**Fig 5:**
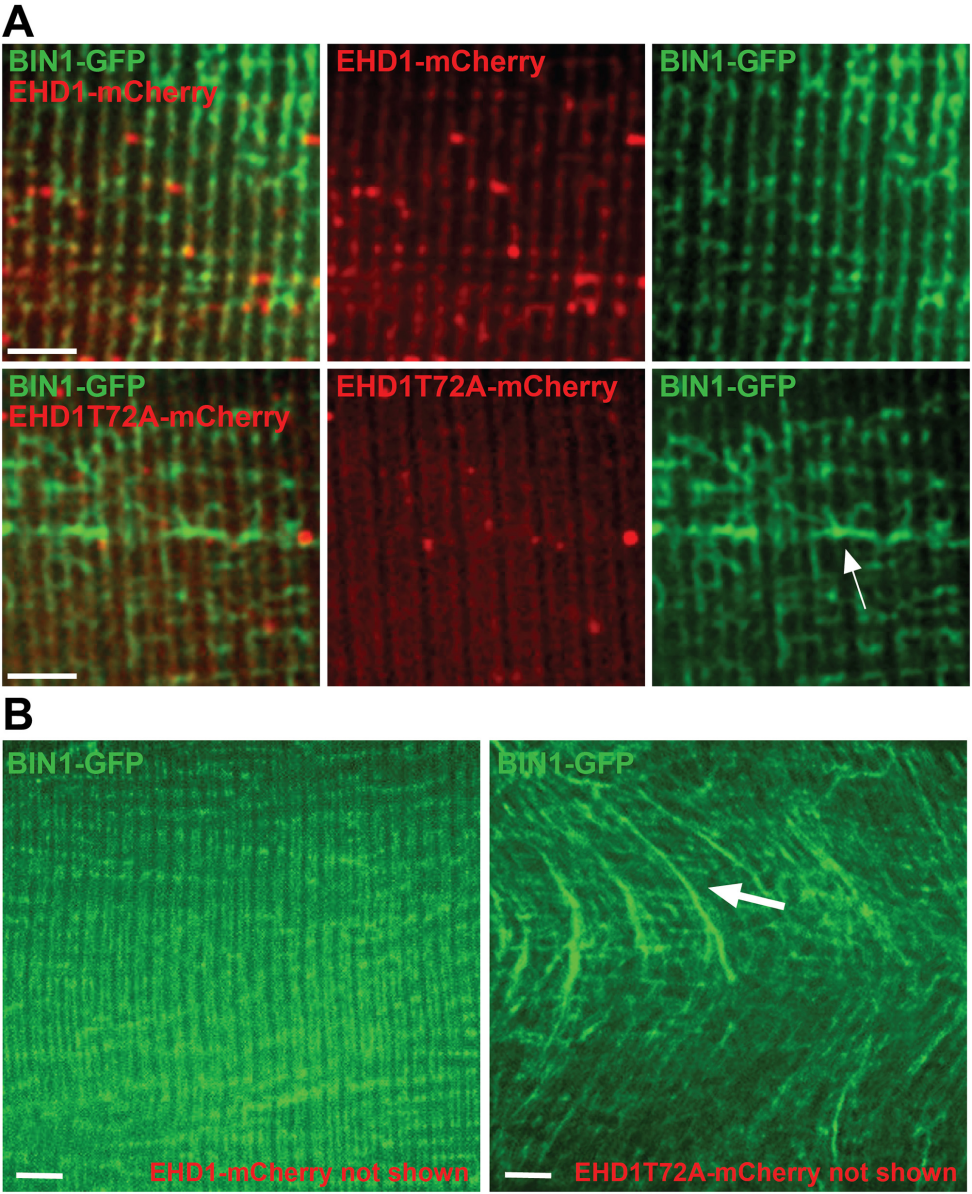
EHD1 modulates BIN1 mediated tubule formation *in vivo*. Myofibers were electroporated with BIN1-GFP and wildtype EHD1-mCherry or EHD1T72A-mCherry. Imaging occurred one week post-electroporation. (A & B) EHD1 and BIN1 normally align in ordered T-tubules in live skeletal muscle. Expression of EHD1T72A results in mislocalization of EHD1T72A and ectopic tubule formation (white arrow), marked with BIN1 staining. Low magnification images are shown below. Scale 5μm. BIN1 mislocalization occurred in 11/11 EHD1T72A myofibers, while 0/11 EHD1 myofibers expressed BIN1 mislocalization.

**Fig 6:**
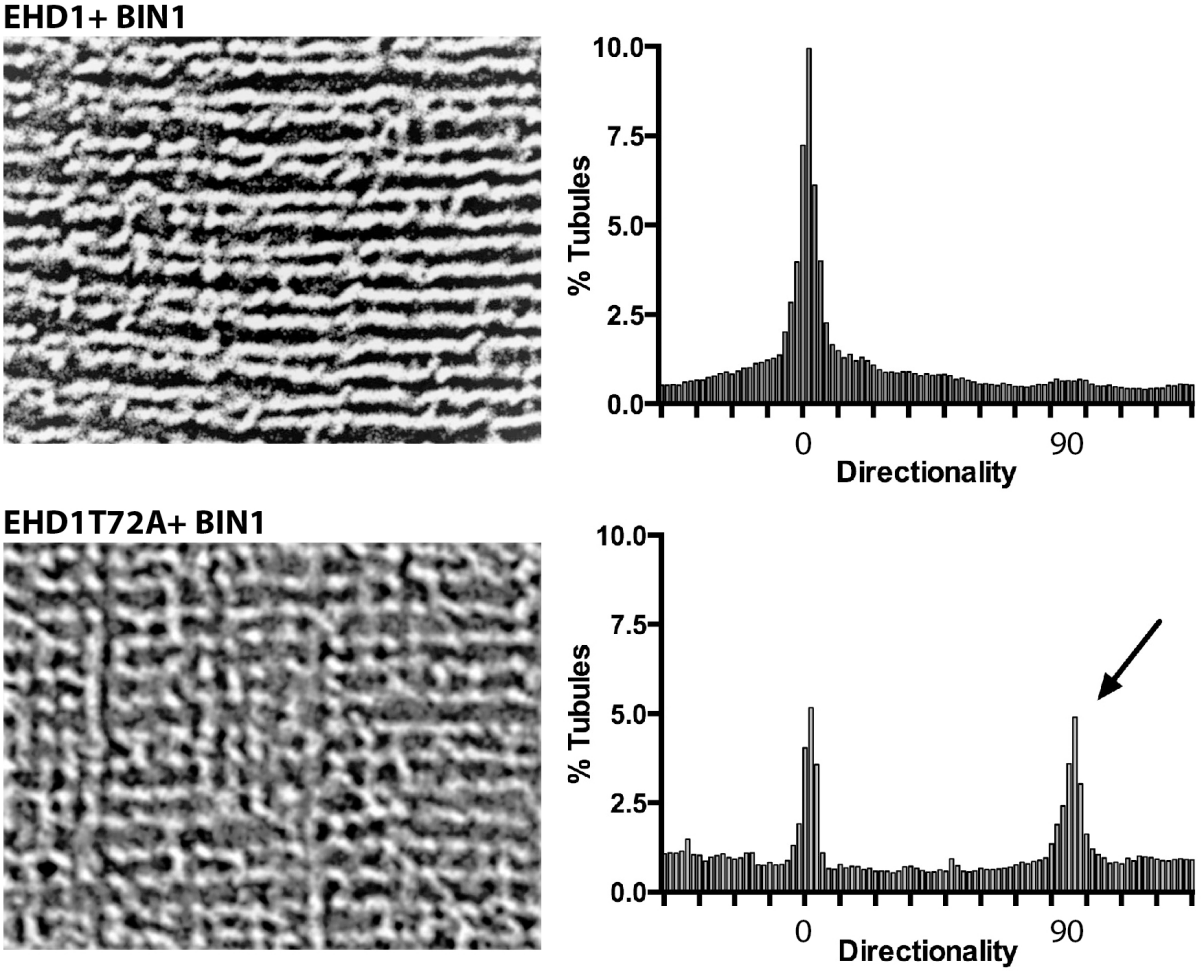
EHD1T72A is a negative regulator of BIN1 mediated tubule formation *in vivo*. Representative images of myofibers coelectroporated with BIN1-GFP and EHD1-mCherry or EHD1T72A-mCherry in wildtype myofibers. Images were processed identically in Fiji and are shown with T-tubules running horizontally. When coelectroporated with EHD1, BIN1 localizes to ordered T-tubules. Graphically this corresponds to the horizontal axis clustering around 0 degrees. Coexpression of EHD1T72A results in mislocalization of BIN1 tubules, causing lateral extensions between longitudinal tubules. Quantification shows a cluster of tubules both at 0 degrees, T-tubules, and at 90 degrees, L-tubules (arrow). Scale 5μm.

To determine if EHD1 modulates mature T-tubules, we conducted *in vivo* electroporation using plasmid expression of EHD1-mCherry or EHD1T72A-mCherry. The T72A substitution abrogates the ATPase activity of EHD proteins, creating a enzymatically dead EHD1 protein [12]. These plasmids were co-electroporated along with a plasmid expressing BIN-GFP into wildtype myofibers, and myofiber imaging was conducted seven days after electroporation to permit time for cell recovery and protein expression. Normally, EHD1 and BIN1 proteins localized in discrete, organized T-tubules throughout the myofiber (Fig 5A, top panel). However, when EHD1T72A was electroporated in conjunction with BIN1, EHD1T72A localized to the T-tubule less efficiently than EHD1 resulting in disrupted BIN1-mediated T-tubule formation. Specifically, expression of EHD1T72A caused ectopic and elongated BIN1-positive tubule formation in myofibers (Fig 5B, white arrow). Abnormal T-tubule formation was assessed using the directionality plugin in Fiji [35–37]. Transverse (T)- tubules are represented at an angle of 0 degrees, while any deviation of this tubule direction is expressed on the histogram accordingly. Electroporation of wildtype EHD1 and BIN1 resulted in tubules plotted only at 0 degrees, indicative of their longitudinal nature. In contrast, EHD1T72A and BIN1 expression resulted in two peaks, one peak at 0 degrees and a secondary peak at 90 degrees indicative of lateral branches developing from the longitudinal tubules in EHD1T72A expressing fibers (Fig 6). A lower magnification image of the enhanced BIN1 tubule formation caused by expression of the dominant negative EHD1T72A mutant is shown in Fig 5B (white arrow). These data suggest EHD1 negatively regulates BIN1 tubule formation *in vivo* in skeletal muscle.

## Discussion

### Reduced EHD1 leads to a reduction in T-tubule remodeling

The EHD1 family of proteins has been implicated in protein-protein interactions necessary for membrane trafficking and especially the formation of intracellular vesicular structures [9,13,38]. BIN1, a BAR domain containing protein implicated in membrane bending, co-immunoprecipitates and colocalizes with EHD1, and EHD1 functions in concert with BIN1 to refine the length and width of membrane tubules [9,13]. Pant et al. used *C*. *elegans* to discover the importance of the BIN1-EHD interaction for endocytic recycling, the process by which vesicles are returned to the membrane. The role of EHDs in trafficking and membrane tubulation was investigated in a cell-based system demonstrating a role for EHD-like proteins in vesiculation and tubulation [38]. We previously co-expressed BIN1 and EHD1 in C2C12 cells, a cell model of muscle development, finding that ectopic intracellular tubules formed in the presence of dominant negative EHD1 and that *Ehd1*-null muscle had T-tubule overgrowth [13]. Each of these cell systems lacks T-tubules, so the current findings in *Ehd1*-heterozygous muscle links partial reduction of EHD1 proteins to the maintenance and remodeling of T-tubules in mature muscle. The association of malformed T-tubules with disrupted expression of Ca^2+^-handling proteins may account for the muscle weakness observed in *Ehd1*-heterozygous muscle. However, alternative mechanisms could lead to muscle weakness, for example the leak of CK from muscle could contribute to altered energy stores (see below).

### T-tubule structure and Ca^2+^ regulation

Efficient muscle contraction requires tight control of intracellular Ca^2+^, which is enabled by an extensive membrane network to trigger Ca^2+^ release throughout the sarcomere [16]. Skeletal and cardiac muscle cells each have deep invaginations of the surface plasma membrane, referred to as the transverse (T-) tubule network. The T-tubules are interwoven with the membranous sarcoplasmic reticulum (SR). Disruption of T-tubule structure is seen in a variety of myopathies, and collectively these disorders have been referred to as “triadopathies” [39]. Modulation of BIN1 expression has been shown to result in impaired Ca^2+^ channel trafficking and aberrant Ca^2+^ transient activity in striated muscle [24,25]. *Ehd1-*heterozygous muscle displayed excessive and disorganized T-tubules, increased BIN1 protein levels, and reduction of junctophilin 2 (JP2) protein expression. Junctophilins directly link the T-tubule and sarcoplasmic reticulum (SR) membranes, anchoring the T-tubule and maintaining the proper spacing and connectivity for normal excitation-contraction (EC) coupling [26]. Genetic loss of JP1 or JP2 results in reduced Ca^2+^ signaling resulting in cardiac failure and embryonic lethality [26,27]. Reduction of JP1 or JP2 in skeletal muscle fibers results in disruption and loss of the triad structure and altered Ca^2+^ release from SR [40]. Furthermore, in cardiomyocytes, JP2 reduction resulted in altered T-tubule orientation promoting an increase in longitudinal tubule formation [41]. We hypothesize that partial loss of EHD1 results in loss of negative regulation of BIN1 activity. With unregulated BIN1 activity, T-tubules extend and grow in an uncoordinated manner, a hypothesis supported by their ectopic position within muscle. The reduction of JP2 protein expression further modulates T-tubules in excess of sarcoplasmic reticulum, and overall disorganized and inadequate triad junction formation. The abnormal morphology of T-tubules, coupled with the marked reduction of JP2, is a muscle substrate that would be expected to display abnormal Ca^2+^ handling properties. Further examination of Ca^2+^ transients, SR release, and store operated Ca^2+^ entry in *Ehd1*-heterozygous myofibers is required.

Dysferlin localizes to the T-tubule specifically at the triad junction, and loss of dysferlin (*Dysf*) results in altered Ca^2+^ homeostasis [22]. Like *Ehd1*-heterozygous mice, *Dysf*-null mice have similar T-tubule abnormalities and DHPR aggregates [22]. We have previously shown that EHD1 weakly binds the C2B domain of DYSF and colocalizes at the T-tubule with DYSF [8,13]. The T-tubule abnormalities in *Dysf*-null mice associate with a reduction in muscle force [31,42]. Due to the co-localization of DYSF and EHD1, we hypothesize that these models share a similar set of defects. Previous studies have also shown a role for BIN1 and DYSF in stabilizing the L-type Ca^2+^ channel, which provides an additional molecular mechanism by which EHD1 deficiency may act [22,24].

### Creatine kinase elevation with abnormal T-tubules

The normal function of CK within a cell is to catalyze the conversion of creatine to form phosphocreatine consuming ATP and generating ADP [43]. In addition to the elongated and ectopic T-tubule, we found markedly elevated serum CK levels in *Ehd1-*heterozygous mice without outward signs of myofiber necrosis or damage. This same finding is seen in caveolin-3-null and young ferlin-null mouse models that show mild changes in histopathology, and T-tubule abnormalities [28,31]. We hypothesize that CK may be transported from muscle into the serum by the disorganized T-tubules. Chou et al. showed human muscle biopsies from individuals with elevated serum CK levels had abnormal tubule structures compared to more than 50 healthy control subjects [44]. Chou concluded that the T-tubule was the primary location resulting in increased CK leak from muscle.

*Ehd1-*heterozygous muscle displays significant weakness compared to wildtype littermate muscle using *ex vivo* force experiments. Similar to *Ehd1*-null mice, *Ehd1*- heterozygous muscle contains primary, structural T-tubule deformities at an early age similar to *BIN1* and *DNM2* mutations, newly subcategorized as “triadopathies” [39]. Functional deficiencies of the T-tubule occur as a secondary consequence in a number of muscle diseases [45–47]. In contrast, there is now an emerging subset of inherited muscle disorders with primary T-tubule and membrane defects, and many of these mutations lead to congenital forms of myopathy. The data presented here indicates that EHD1 is implicated in T-tubule formation and function, and suggests that EHD1 is a potential candidate gene for unresolved genetic myopathy.

## Materials and Methods

### Animals

The *Ehd1*-null allele was previously generated by deleting exon 1 [20]. Mice were maintained on a mixed C57Bl/6 and 129Sv background, and housed in a specific pathogen free facility in accordance with Institutional Animal Care and Use Committee (IACUC) regulations. Euthanasia was performed through carbon dioxide or anesthetic gas inhalation followed by cervical dislocation and removal of the heart. All methods using living animals in this study were performed in ethical accordance with the American Veterinary Medical Association (AVMA) and under protocols fully approved by both the Institutional Animal Care and Use Committee (IACUC) at the University of Chicago (protocol 70619) and the IACUC at Northwestern University Feinberg School of Medicine (protocol number ISO00000911). Consistent with the approvals stipulated by these protocols, all efforts were made to minimize suffering.

### Muscle analysis

Quadriceps and triceps muscles were dissected, formalin fixed and embedded in paraffin. Sections from the mid-belly of the muscle were stained with hematoxylin and eosin. Using Image J, the mean fiber cross sectional area was calculated by counting individual fibers within at least five random images from at least three *Ehd1-*heterozygous and two wildtype mice. Statistics were performed with Prism (Graphpad, La Jolla, CA) using an unpaired t-test.

### Immunoblotting

Proteins in lysates of 2-3month old wildtype and *Ehd1-*heterozygous quadriceps muscles, (n ≥ 2), were transferred to membranes and were immunoblotted with anti-BIN1 (1:1000, sc-23918, Santa Cruz, Dallas, TX), rabbit polyclonal anti-EHD1 (1:5000, [10]), anti-DHPR (1:1000, ma3-920, Thermo Scientific, Rockford, IL), anti-Junctophilin 2 (1:1000, sc-51313, Santa Cruz, Dallas, TX), anti-Junctophilin 1 (1:1000, 40–5100, Thermo Scientific, Rockford, IL) antibodies. Secondary antibodies, goat anti-rabbit, and goat anti-mouse conjugated to horseradish peroxidase (Jackson ImmunoResearch) were used at a dilution of 1:2500. Blocking and antibody incubations were done in Starting Block T20 (Invitrogen, Grand Island, NY) for all antibodies and rinsed with TBS-T. Memcode and gelcode stains were used to mark proteins for loading controls (Life Technologies, Grand Island, NY). Chemiluminescence substrate, Kodak Biomax MS film, and a UVP BioSpectrum Imaging System (Upland, CA) were used for detection.

### Muscle Preparation and Mechanics

Intact *extensor digitorum longus* (EDL) muscles from 8-week-old male mice were excised in regular rodent Ringer’s solution (in mM: 146 NaCl, 5 KCl, 2 CaCl_2_, 1 MgCl_2_, and 10 HEPES, pH 7.4) essentially according to the Treat-NMD standard operating procedure. Muscles were suspended in a vertical tissue bath containing oxygenated Krebs solution (in mM: 121 NCl, 5 KCl, 1.8 CaCl_2_, 0.5 MgCl_2_, 0.1 EDTA, 0.4 NaH_2_PO_4_, 24 NaHCO_3_, and 5 glucose, pH 7.4 with continuous bubbling 95% O_2_/5% CO_2_) and maintained at 25°C. The isolated muscles were stimulated with 0.5 ms pulses at 1 A via two parallel platinum electrodes flanking the muscle. The length of each EDL was adjusted to that which produced the maximal twitch force (L_0_), which was measured using fine calipers. Maximal twitch and tetanic force were elicited with a series of three twitch/tetanus trains given 3 minutes apart, each train consisting of a single twitch followed by 10 secs rest then a 500-ms/150-Hz tetanus. At the conclusion of each experiment, each muscle was blotted and immediately weighed. All data were analyzed offline following data import into Clampfit software (Molecular Devices, Clampfit v10.2.0.14). The magnitude of isometric twitch and tetanus were determined as the peak twitch and peak tetanus force. All peak force measurements were normalized to cross-sectional area and expressed as specific force (sP_0;_ mN/mm^2^). Statistical significance was determined by t-test using Prism software.

### Electron microscopy and Tubule Analysis

Quadriceps muscles from 2-month old wildtype and *Ehd1-*heterozygous mice were dissected. To visualize the T-tubules, muscles were fixed in 2.5% glutaraldehyde with 75 mM calcium chloride in 0.1mM sodium cacodylate for 3 hours, postfixed in 2% osmium tetroxide containing 0.8% potassium ferricyanide for 2 hours at 4°C, rinsed, dehydrated in ethanol, and embedded in epoxy resin modified from [29]. Samples were sectioned and stained with 1% uranyl acetate followed by lead citrate. Images were taken on a Philips CM10 electron microscope. Analysis was performed on 2D images. T-tubules were scored abnormal if ectopic duplicated or longitudinal in orientation. Three mice per genotype were analyzed equaling a total of greater than 400 structures per genotype. Statistics were performed with Prism (Graphpad, La Jolla, CA) using an unpaired t-test.

### Evans Blue Dye Assay

As described previously in [48]. Briefly, Evans blue dye (E-2129, Sigma) was dissolved in phosphate buffered saline (PBS) at 10 mg/ml. Each animal received an intraperitoneal injection of dye at 5 μl/g body weight. Approximately 48 hours after injection, tissues were harvested, finely minced, weighed and incubated at 55°C in 1ml of formamide for 2 hours with shaking. Spectrophotometric absorbance was measured at 620 nm. Statistics were performed with Prism (Graphpad, La Jolla, CA) using an unpaired t-test.

### Serum Biomarker Assay

Age-matched wildtype and *Ehd1-*heterozygous blood samples were collected from the vasculature of pups sacrificed by cervical dislocation (P0) or eye bleeds (8wk, 6m, 14m) using heparin-treated capillary tubes (Fisher, Pittsburgh, PA) into serum separator tubes (Becton Dickinson, Franklin Lakes, NJ) and centrifuged for 10 min at 8000 *g*. The plasma fractions were frozen and stored at −80°C. Creatine kinase activity was determined with the EnzyChrom CK Assay kit (BioAssay Systems, Hayward, CA) and a FluoStar Optima plate reader (BMG Labtech, Cary, NC). Statistics were performed with Prism (Graphpad, La Jolla, CA) using an unpaired t-test.

### Plasmids and Electroporation

EHD1-mCherry and EHD1 T72A-mCherry were described previously. pcDNA3 EHD1-GFP and EHD1 T72A-GFP were generated by subcloning a carboxy-terminal EGFP tag into EHD1 or EHD1T72A pcDNA3 using BamH1 and Xho1 restriction enzymes (New England Biolabs, Ipswich, MA). pEGFPC1-muscle Amphiphysin II (BIN1 variant 8) was purchased from Addgene (Cambridge, MA). FDB fibers were transfected by the *in vivo* electroporation methods described in detail in [49]. Muscle fibers were isolated as described and studied seven days after electroporation to allow for recovery and protein expression in the electroporated muscles. Images were acquired on the Leica SP5 II STED-CW super resolution laser scanning confocal microscope in conventional mode.

### FDB preparation and Immunofluorescence Microscopy

The flexor digitorum brevis (FDB) muscle bundle was dissected and placed in 1ml of DMEM containing BSA plus collagenase solution pre-warmed to 37°C in a 12-well plate. After 2 hours, fibers were triturated in Ringers solution. Fibers were fixed on coverslips with 4% paraformaldehyde blocked in 1X phosphate-buffered saline (PBS) containing 10% fetal bovine serum and triton, and then immunostained at 1:100 with anti-DHPR (ma3-920, Thermo Scientific, Rockford, IL) and at 1:100 with anti-BIN1(sc-30099, Santa Cruz, Dallas, TX). The anti-EHD1 rabbit antibody was previously described [10]. Goat anti-rabbit conjugated to Alexa 488 and goat anti-mouse 594 were used at 1:2000. Slides were mounted with Vectashield with DAPI. Images were captured using a Leica SP5 II STED-CW super resolution laser scanning confocal microscope in standard mode.

### Immunofluorescence Microscopy Tubule Analysis

Images were acquired as above and Fiji was used for the following steps. Images were rotated such that the Transverse (T)-tubules were orientated in the horizontal plane. Images were manipulated identically as follows: background fluorescence was subtracted, threshold was adjusted and then the directionality plugin was run. 0 degrees is equivalent to the transverse-tubule, while 90 degrees is equivalent to the longitudinal-tubules [35–37]. Histograms were generated in Prism (Graphpad, La Jolla, CA).

## Supporting Information

S1 Fig Decreased EHD1 expression and reduced myofiber size in *Ehd1-*heterozygous mice. (A) Muscle lysates were prepared from 2-3m old WT and *Ehd1+/-* quadriceps muscle and immunoblotted with anti-EHD1. *Ehd1*+/- muscle showed a 60% reduction in EHD1 protein expression levels p = 0.003 (n = 4 per genotype). Gel code is shown as a loading control (LC). (B) *Ehd1+/-* triceps muscle shows smaller myofibers and myofiber splitting (long arrow) at 8-weeks of age by H&E staining. (C) *Ehd1+/-* fibers have reduced mean cross sectional area (CSA) compared to WT controls at 8-weeks (n>500 fibers, p = 0.008). (D) Histogram showing the shift (green arrows) in myofiber CSA in *Ehd1+/-* muscle at 8-weeks (n>500 fibers, p<0.001). (E) Hallmark signs of dystrophy were lacking in 14-month old WT and *Ehd1*-*heterozygous* (*Ehd1+/-)* muscle stained with hematoxylin and eosin. Scale 50μm.
(TIF)

S2 Fig Normal Evans Blue Dye uptake in *Ehd1*-heterzygous muscle tissues. EBD was injected into 8-week-old WT and *Ehd1-*heterozygous (*Ehd1+/-)* mice. Forty-eight hours post injection tissues were harvested and analyzed for EBD uptake (expressed as absorbance per mg of tissue). The level of EBD uptake was non-significant for all muscles analyzed between WT and *Ehd1+/-* (n ≥ 6 for both genotypes).
(TIF)
