# Supplementary figures and images for: Eps 15 Homology Domain (EHD)-1 Remodels Transverse Tubules in Skeletal Muscle
Source: PLoS One. 2015 Sep 1;10(9):e0136679. doi: 10.1371/journal.pone.0136679 (PMC4556691; doi:10.1371/journal.pone.0136679)

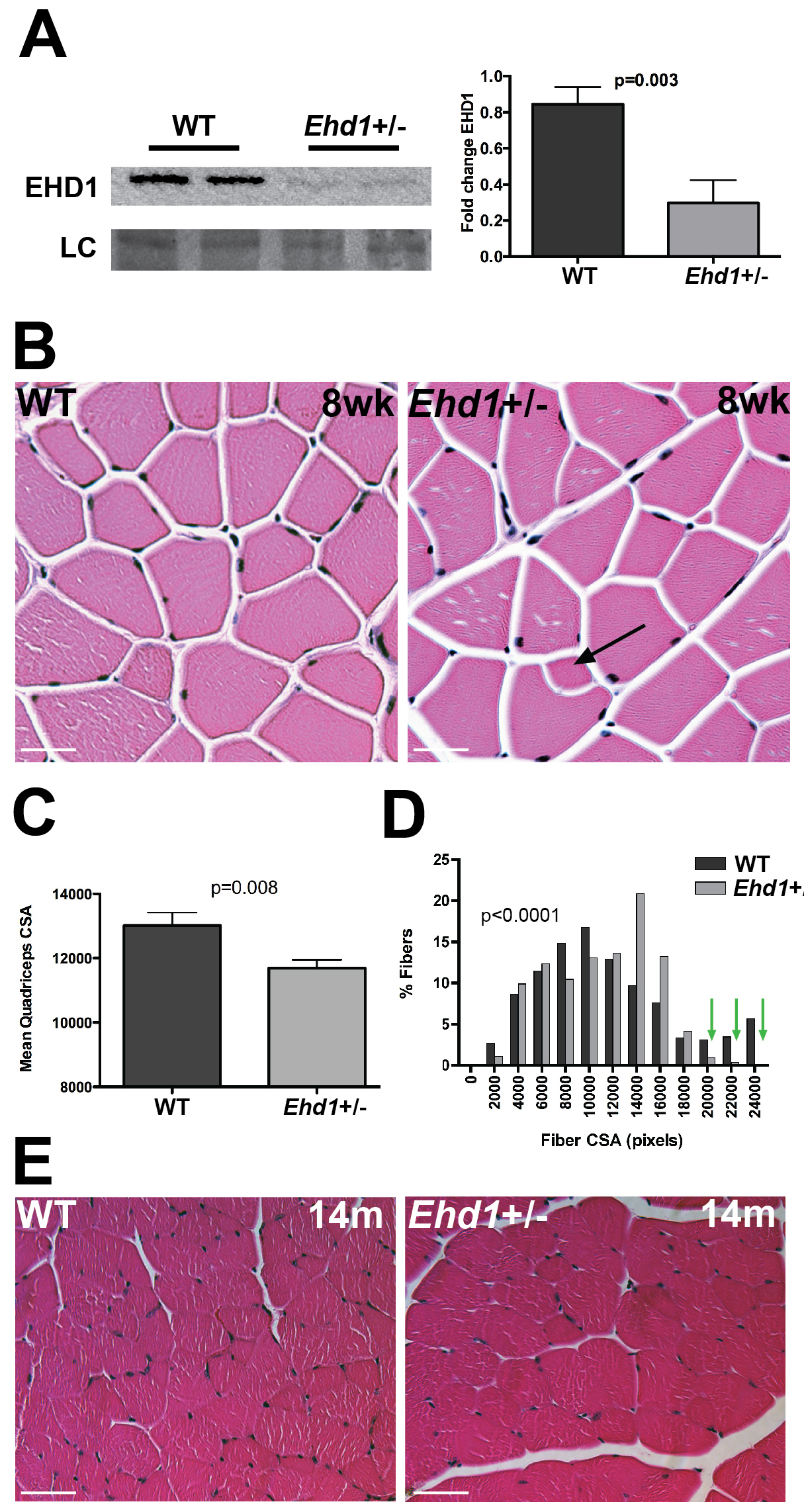

Supplement: S1 Fig — (A) Muscle lysates were prepared from 2-3m old WT and Ehd1+/- quadriceps muscle and immunoblotted with anti-EHD1. Ehd1+/- muscle showed a 60% reduction in EHD1 protein expression levels p = 0.003 (n = 4 per genotype). Gel code is shown as a loading control (LC). (B) Ehd1+/- triceps muscle shows smaller myofibers and myofiber splitting (long arrow) at 8-weeks of age by H&E staining. (C) Ehd1+/- fibers have reduced mean cross sectional area (CSA) compared to WT controls at 8-weeks (n>500 fibers, p = 0.008). (D) Histogram showing the shift (green arrows) in myofiber CSA in Ehd1+/- muscle at 8-weeks (n>500 fibers, p<0.001). (E) Hallmark signs of dystrophy were lacking in 14-month old WT and Ehd1-heterozygous (Ehd1+/-) muscle stained with hematoxylin and eosin. Scale 50μm. (TIF) [file pone.0136679.s001.tif]

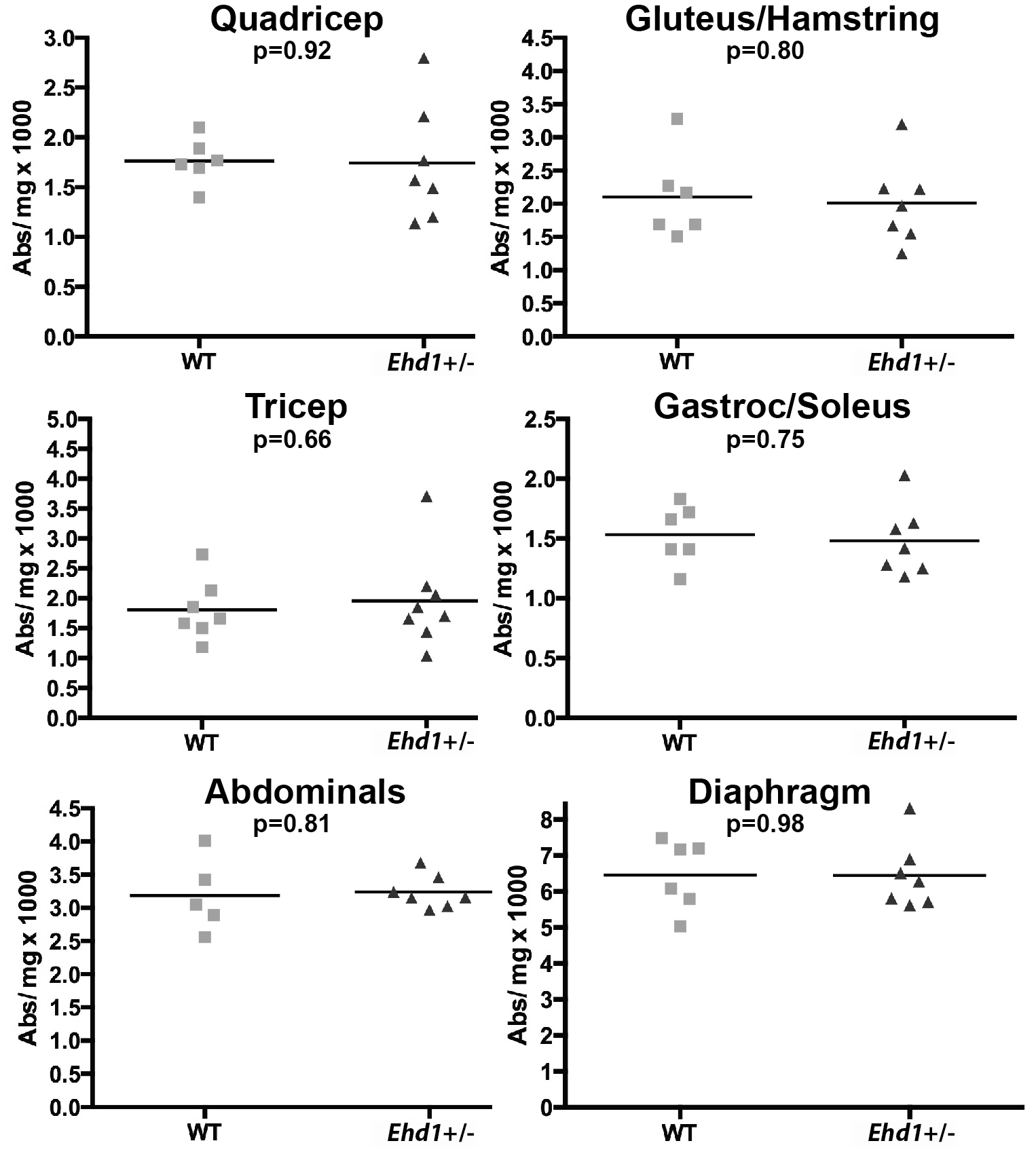

Supplement: S2 Fig — EBD was injected into 8-week-old WT and Ehd1-heterozygous (Ehd1+/-) mice. Forty-eight hours post injection tissues were harvested and analyzed for EBD uptake (expressed as absorbance per mg of tissue). The level of EBD uptake was non-significant for all muscles analyzed between WT and Ehd1+/- (n ≥ 6 for both genotypes). (TIF) [file pone.0136679.s002.tif]
